# Corrigendum

**DOI:** 10.1002/vms3.1095

**Published:** 2023-02-09

In the article by Hamdy et al. (2023), the 6^th^ author's surname was misspelled. It should read as “Shaalan” instead of “Shalaan”.
